# Effects of computer-based cognitive training vs. paper-and-pencil-based training on the cognitive development of typically developing children: Protocol for a randomized controlled trial

**DOI:** 10.1016/j.mex.2024.102877

**Published:** 2024-07-23

**Authors:** Carolina Robledo-Castro, Gimena Rocío Ramírez-Suarez, Luz Helena Rodríguez-Rodríguez

**Affiliations:** University of Tolima, Santa Helena alta Cl 42 1-02, Ibagué 730006299, Colombia

**Keywords:** Cognitive training, Executive functions, Attention, Computer-based, Cognitive development, Randomized controlled trial to evaluate the effect of a cognitive training program

## Abstract

The research aims to evaluate the effectiveness of a computerized cognitive training program in improving executive functions and attention in elementary school children, compared to a traditional paper-and-pencil intervention. The study has been formulated as a randomized controlled trial with pre- and post-intervention measures. For the study, third-grade children with typical development aged between 7 and 9 years will be recruited. Participants will be randomly assigned to the two study arms (control and experimental). The experimental group will participate in a computerized intervention using the NeuronUp cognitive stimulation platform for 8 weeks, twice a week. Sessions will be conducted using computers in the computer lab of the participating educational institution. The active control group will engage in paper-and-pencil cognitive training for the same duration and intensity as the experimental group. Evaluators will be blinded to the assignment, and participants will be blinded to the target intervention. Hypothesis testing will be conducted through ANOVA-MR, and logistic regressions will be implemented to assess the effect of socioeconomic variables on children's performance. These results are expected to contribute to the discussion on the opportunities and benefits offered by cognitive training programs on the cognitive development of typically developing children.

## Graphical abstract

**Figure:**
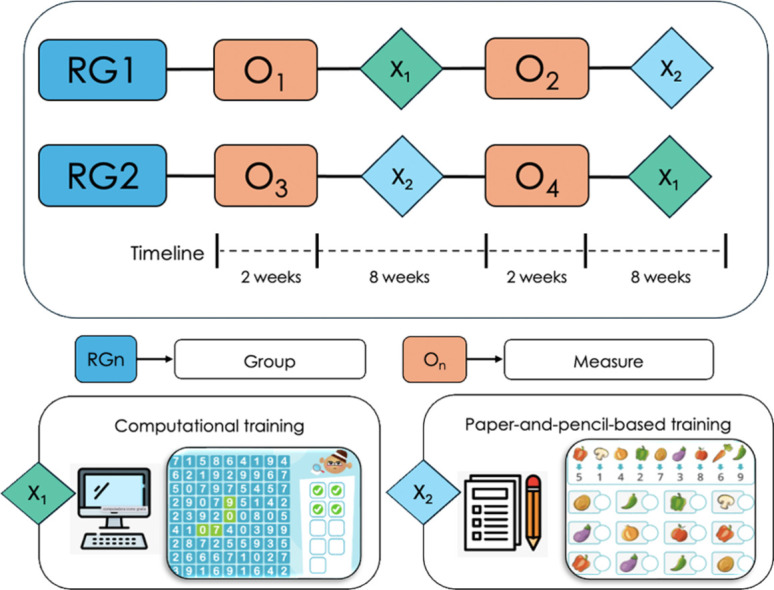

Specifications table

**Table:** 

Subject Area:	• Cognitive sciences
More specific subject area:	• Children's Cognitive Development
Name of your method:	Randomized controlled trial to evaluate the effect of a cognitive training program
Protocol name:	Effects of computer-based cognitive training vs. paper-and-pencil-based training on the cognitive development of typically developing children: Protocol for a randomized controlled trial.
Reagents/tools:	• Computer-based cognitive training • Paper-and-pencil cognitive training
Experimental design:	A randomized controlled trial with intra-subject and inter-subject evaluations will feature two parallel groups: an experimental group and an active control group. Pre-test and post-test measures will be conducted. Participants will consist of two third-grade classes, aged 7 to 10 years (*N* = 58). Random allocation will be employed. Primary measures will include neuropsychological tests to assess executive functions (cognitive flexibility, working memory, inhibitory control, planning, and attention). The experimental group will undergo computer-based cognitive training, while the control group will undergo paper-and-pencil cognitive training. The intervention will take place at the educational institution, twice a week in 40-minute sessions, spanning 8 weeks with a total of 16 sessions. Evaluators responsible for pre-test and post-test measurements will be blinded to the allocation. Researchers in charge of the intervention will be blinded to the evaluation results.
Trial registration:	The study was registered in the Office of Research and Scientific Development at University of Tolima under code 50,119.
Ethics:	The randomized controlled trial complies with the ethical parameters defined by the Helsinki Declaration [1], as well as the norms for health research established by the Colombian Ministry of Health [2] in Resolution 8430. Parents and guardians of the children who agree to participate will sign informed consent forms. This randomized controlled trial has been approved by the bioethics committee of University of Tolima, as documented in minutes No. 10 of December 13, 2022.
*Value of the Protocol:	• This protocol is important for verifying the effects of computer-based cognitive training on the development of executive functions in typically developing school-aged children. • This protocol presents a systematic proposal for computer-based training that could contribute to the design of new programs in similar populations. • This protocol presents the necessary methodological elements to facilitate its pre-application in future studies.

## Background

Computer-based cognitive training programs have gained popularity in recent years, offering several advantages over traditional pencil-and-paper interventions. However, there is a strong debate about the effectiveness of computer-based cognitive training programs and the long-term permanence of their effects [3]. This debate has led to positions both for and against the benefits of these programs. Studies suggest that computer-based cognitive training programs produce near transfer effects on cognitive functions, with less evidence for far transfer [4]. Overall, numerous studies have shown encouraging results regarding the improvement of specific cognitive skills and their applicability in daily life and academic settings [5]. However, the effects of introducing these types of programs in school settings with typically developing children are still relatively understudied.

A general review of the literature shows that computer-based cognitive training programs have demonstrated their effectiveness in improving cognitive functioning across multiple dimensions, with compelling results in various populations. For instance, cognitive training programs have shown good results in different populations such as children with learning difficulties or neurodevelopmental disorders [6], and improvements in working memory, attention, and academic performance in children with ADHD [[7], [8], [9], [10]]. Additionally, these programs have led to improvements in executive functions (EF) in children with behavioral, emotional, and social difficulties [11], non-verbal memory in children with intellectual disabilities [12], and reading skills in children with special educational needs [13]. Furthermore, training of working memory has been associated with achievements in mathematics and reading [[5], [14],^15^]. Cognitive training has also been beneficial for adolescents with anxiety and low attentional control, for improving executive functions in preschool children [16], and for children with working memory difficulties [6,17]. Finally, training of executive functions has been effective in neurotypical children [18].

Cognitive training has been widely studied in recent years, and it has been concluded that the success of interventions largely depends on the study design, the training program, the target group, and the selected outcome measures [19]. Important aspects to consider in this type of study include the number of participants, the type of instruments selected to assess the impact of training, the inclusion of an active control group, random assignment of groups [6], and control of study bias risks [20]. In other words, the rigor with which the study is formulated and conducted is crucial.

Given this background, there is a clear need for study protocols with methodological rigor to ensure that the evidence collected on the effects of computerized and paper-and-pencil cognitive training is valid and reliable. Additionally, a rigorous methodological design allows for the study to be replicable in other contexts. The current study protocol was designed in response to this issue.

## Description of protocol

There is strong evidence showing that developmental contexts and human experiences during childhood are determinants of cognitive development [21,22]. Similarly, it has been demonstrated that the family and school environment in the early years of life have a key impact on the development of various cognitive domains, as well as on the function and structure of the brain networks that support them [[23], [24], [25]]. Additionally, it has been observed that children in more vulnerable conditions tend to have poorer performance in their cognitive processes, which influences their academic outcomes [[23], [26], [27]].

These findings have highlighted the need to provide developmental and learning opportunities for children throughout their growth, promoting their cognitive skills [[19], [22]]. This has been a significant challenge in both the fields of cognitive sciences and human development and education. In response to this issue, various intervention programs have emerged in recent years aimed at children with neurodevelopmental disorders as well as typically developing children, such as cognitive training programs. As a result of the emergence of these programs, there has also been a growing number of studies aimed at examining the impact of training programs on children's cognitive abilities to identify the benefits, limitations, and opportunities for improvement of existing programs [[5], [28],^29^]. The present protocol showcases research that seeks to contribute to this developing field of study.

## Cognitive training and brain plasticity

Cognitive stimulation refers to a set of techniques and strategies aimed at improving overall cognitive performance or specific processes and components (such as attention, memory, language, executive functions, and calculation), whether in healthy individuals or in patients with alterations in their central nervous system [19]. In childhood, cognitive stimulation aims to provide children with experiences that support cognitive development in domains that are still evolving, thus promoting learning that the child has not yet acquired [30].

Cognitive stimulation is guided by the principles of transfer and generalization [31] and is supported by mechanisms of brain plasticity—the brain's ability to change in response to experience or environmental stimulation [21]. Brain plasticity facilitates processes such as reorganization of functional interactions among different neuronal groups, the incorporation of new areas into previously established networks, and neuronal adaptability in areas adjacent to affected brain regions [32,33]. While many cognitive stimulation programs targeted at children have been developed in clinical settings, interventions in school and home environments are gaining increasing recognition.

## Computerized programs and paper-and-pencil programs

Cognitive training consists of a series of activities and exercises selected to harness the brain's plasticity, providing individuals with challenging situations that promote new learning and strengthen problem-solving skills. There are essentially two types of cognitive training programs: process-based training and strategy-based training [29]. Process-based training involves repeatedly performing cognitive tasks, typically with increasing difficulty as the individual progresses. This progressive complexity ensures a constant stimulus that promotes cognitive development [17].

Many of these tasks are often based on classical training paradigms such as n-back, go/no go, and the Flanker task, among others [34]. The goal of this type of training is to improve specific functions so that they can be transferred and applied to real-life problems.

On the other hand, strategy-based training teaches procedures and strategies to improve cognitive functions [29], including metacognitive strategies, reading techniques, and self-instructional training, among others.

The advancement of digital technologies has led to the emergence of assistive technologies for cognitive stimulation and rehabilitation, introducing new approaches that have propelled the popularity of computerized cognitive training over traditional paper-based methods. Programs supported by digital interventions, often referred to as 'cognition-supporting technologies,' have shown significant impacts on training various processes such as verbal communication, executive functions, and social participation [[35], [36]]. These programs have evolved from first-generation games and activities to fourth-generation technology today [8,37].

Computerized cognitive training offers several advantages over paper-and-pencil methods, primarily due to the customization and adaptability provided by software. These programs are often more interactive and visually appealing, providing instant feedback on performance and enhancing accessibility across different environments, thereby integrating seamlessly into the subject's routine. They also enable monitoring and tracking of progress, offer a wide variety of tasks, and incorporate more sensory modalities compared to paper-and-pencil activities [34].

Although computerized cognitive training offers these advantages, it does not necessarily completely replace paper-and-pencil training, which can be particularly useful when technology is unavailable, for promoting fine motor skills, or simply to provide variety in training modalities. The choice between one method or another (or a combination of both) may depend on individual preferences, specific training objectives, and the learning context.

The choice of cognitive training as part of a clinical or educational intervention program must be supported by solid evidence. To contribute to the evidence on the uses, scope, and limitations of cognitive training programs, including the comparison between computerized and paper-and-pencil modalities, we have designed a randomized controlled trial protocol. This protocol aims to compare the effects of computerized cognitive training with pen-and-paper activity-based training on various executive cognitive processes (such as working memory, cognitive flexibility, inhibitory control, and planning) as well as basic cognitive processes like attention.

The protocol was developed following the standards and rigor set by the Consolidated Standards of Reporting Trials (CONSORT), which aim to ensure accurate and transparent description of the design, execution, and data analysis of all randomized controlled trials. It also adheres to ethical standards established for studies involving human subjects. The protocol includes the methodological design of the study and provides detailed descriptions of the interventions studied, facilitating replication in future research. Additionally, it incorporates measures to control biases, ensuring the quality and robustness of the collected evidence.

## Specific objectives and hypotheses

The current randomized controlled trial (RCT) aims to evaluate the effect of computerized cognitive training on the executive functions of third-grade neurotypical children, aged 7 to 10 years, compared to paper-and-pencil cognitive training. In response to these research objectives, the following working hypotheses have been formulated:

- Null Hypothesis (H_0_): There are no significant differences in executive functions between children receiving computerized cognitive training and those receiving paper-and-pencil cognitive training.
- Alternative Hypothesis 1 (H_1_): It is expected that the group of children receiving computerized cognitive training will show greater improvements in their executive functions compared to those receiving paper-and-pencil cognitive training.
- Alternative Hypothesis 2 (H_2_): It is expected that the group of children receiving paper-and-pencil cognitive training will show greater improvements in their executive functions compared to those receiving computerized cognitive training.

## Protocol design

**Fig. 1:**
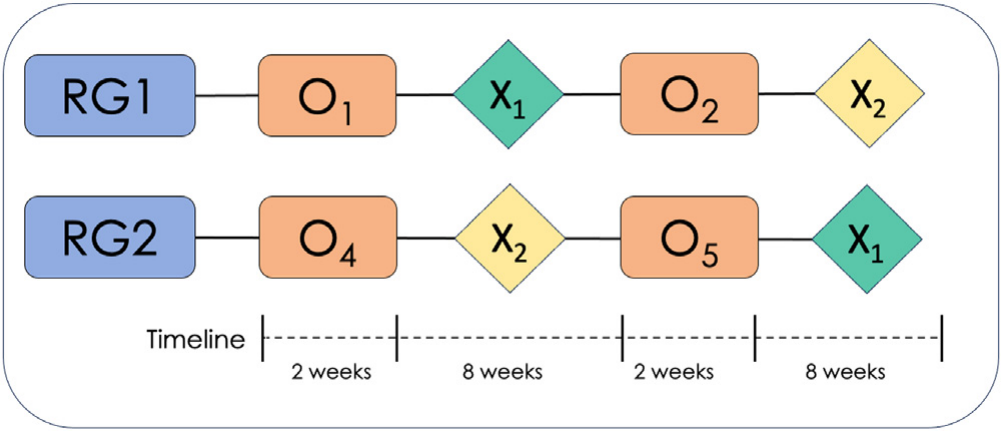
Experimental design scheme.

*Note:* RG1: Experimental group; RG2: Control group; O_n_: Measurement; X_1_: Experimental intervention; X_2_: Control intervention.

The study is designed as a randomized controlled trial with intra-subject and inter-subject evaluations, employing a crossover design involving two simultaneous groups: an experimental group undergoing computerized cognitive training and an active control group undergoing traditional paper-and-pencil cognitive training. Two measures will be administered—one before the intervention (pre-test) and another after the intervention (post-test). Subsequently, following the post-test, the groups will switch interventions. The clinical trial protocol adheres to the CONSORT guidelines [38]. Fig. 1 illustrates the experimental design scheme.

## Criteria for inclusion and exclusion

The target population for the study consists of third-grade children from public elementary schools in Colombia. The following inclusion criteria have been established: 1) Children of both sexes; 2) Currently enrolled in the third grade of elementary school; 3) Aged between 7 years 0 months and 9 years 11 months at the beginning of the study.

The exclusion criteria are defined as follows: 1) Children with uncorrected sensory disabilities that limit their participation in intervention activities; 2) Children diagnosed with psychiatric disorders or neurodevelopmental disorders.

## Sample size calculation

A projected estimate was conducted using G-Power to establish parameters for sample size calculation (see Table 1), based on previous studies that reported moderate to large effect sizes in similar research [39]. This estimation suggests a group size of 29 children, resulting in a total sample size of 58 children.

**Table 1:** Parameters for group size calculation.

Parameter	Value
Effect size	0,75
α confidence level	0,05
Sample power	0,8
Number of groups	2
Number of measures	2
Total sample size	*N* = 58
Sample size per group	*N* = 29

## Allocation

The allocation of subjects to groups will be randomized. Randomization will be conducted by an investigator not involved in the study, using the OxMaR Spanish version tool for clinical trial randomization developed by Guillaumes & O'Callaghan [40].

## Outcome measures

The executive functions of the children will be assessed using neuropsychological tests that have established validity and reliability for this population and within the context of such studies. The selection of tests for this protocol was based on recommendations from Robledo et al. [39]).

• **Planning**

To assess planning, the Tower of London test has been selected. This cognitive task, originally designed by Tim Shallice in 1982 and later constructed and validated by Culbertson & Zillmer [41], evaluates executive functions, particularly planning and problem-solving. The test requires participants to move a series of colored disks between three poles to achieve a specific configuration using the fewest possible moves. The task includes constraints such as limits on the number of disks that can be moved at once and rules prohibiting larger disks from being placed on top of smaller ones.

• **Working memory**

Since working memory comprises various components, different measures were selected to assess each process. For verbal-auditory working memory, the Digit Span subtest of the WISC-V (Wechsler Intelligence Scale for Children, Fifth Edition) was chosen. This subtest includes two main parts: Forward Digit Span and Backward Digit Span [42]. In the Forward Digit Span task, the evaluator reads a series of numbers aloud at a steady pace, and the child repeats them in the same order they were presented. The length of the number sequences progressively increases from shorter to longer sequences. In the Backward Digit Span task, the procedure is similar, but the child is required to repeat the numbers in reverse order.

Spatial working memory will be assessed using the Corsi Block-Tapping Task, a component of the Wechsler Memory Scale III battery [43]. This task involves a set of identical blocks arranged in fixed positions with irregular distances between them [44]. During the task, the evaluator taps the blocks in a specific sequence, which the participant must then reproduce by tapping the blocks in the same order they were presented. The sequence length gradually increases throughout the task. Similar to the Digit Span test, the Corsi Block-Tapping Task includes two parts: in the first part, the participant taps the blocks in the same order as presented, and in the second part, they tap the blocks in reverse order.

For visual working memory, the Visual Span subtest of the WISC-V test battery [42] will be used. In this task, the child is shown a series of images in a specific order. Subsequently, a larger group of images is presented, and the child must identify the images and their sequence from the initial presentation. The number of images progressively increases throughout the task.

• **Inhibitory control and cognitive flexibility**

To assess inhibitory control and cognitive flexibility, the classic Stroop test task was selected [45]. The Stroop Test evaluates interference control, mental flexibility, and attentional management. The test is based on the interference phenomenon, which occurs when processing one feature of a stimulus interferes with processing another. In the first part of the task, the subject is given a list of color words (blue, red, green) written in black ink and must read them in order. Next, a list of stimuli consisting of three letters 'X' printed in different colors is presented, and the subject must name the color of the ink. Finally, a list of color words (blue, red, green) printed in various colors is shown, and the subject must indicate the color of the ink without reading the word. The subject has 45 s to complete each list.

Another measure to assess cognitive flexibility will be the Wisconsin Card Sorting Test (WCST), a neuropsychological assessment tool that evaluates planning ability, cognitive flexibility, abstract reasoning, and problem-solving. A shortened version of 48 cards has been validated for use in children [46]. In this test, the subject must sort a series of cards that vary in color, shape, and number of figures. The goal is for the participant to identify a sorting rule that is not explicitly stated and that changes throughout the test. The challenge lies in the individual's ability to detect changes in the sorting rule and adapt to them flexibly.

• **Attention**

For attention, two neuropsychological tests were selected: the d2 Attention Test and the Trail Making Test. The d2 Attention Test, developed by Rolf Brickenkamp [47], measures sustained attention and processing speed. It consists of a worksheet with several rows of the letters 'd' and 'p,' each accompanied by one, two, three, or four small dashes above or below. The task requires the participant to cross out all the 'd' letters with two dashes above or below, ignoring all other letters and marks. The activity is timed, with a 20-second limit for each row, and includes a total of 20 rows.

The Trail Making Test (TMT) [48] evaluates visual processing speed, sustained attention, and divided attention. The test consists of two parts: Part A involves a sheet with randomly distributed numbers that the subject must connect in ascending order as quickly as possible. Part B is similar to Part A but includes both letters and numbers. The subject must connect them in ascending order while alternating between numbers and letters [49]. For this study, the children's version of the TMT, which has fewer stimuli, will be used.

• **Covariate measures**

Sociodemographic data for each participant will be collected through a sociodemographic characterization questionnaire. This questionnaire includes variables such as parents' level of education, access to technological and informational resources, family socioeconomic status, and family composition, among others. These data will be treated as covariates to observe the correlation of these variables with the performance of the participating children, thereby achieving better control of confounding variables.

## Interventions

• **Experimental group**

The experimental group will participate in computerized cognitive training. The intervention will take place in the computer lab of the participating educational institution. Each session will last 40 min, with a frequency of two times per week, for a duration of eight weeks, totaling 16 sessions.

**Fig. 2:**
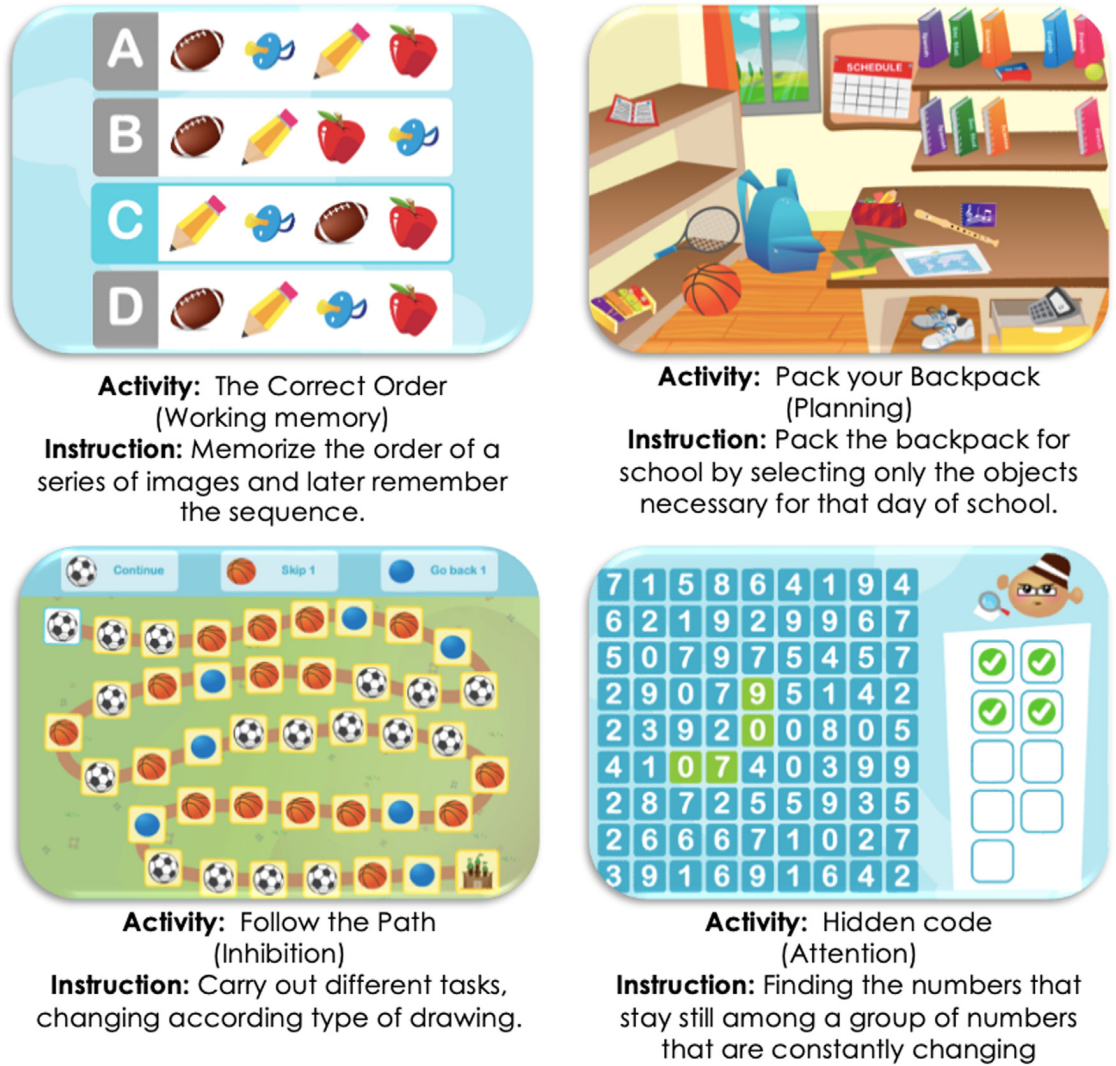
Example activities from NeuronUP.

***Source***: personal compilation based on www.neuronup.com.

For the development of the intervention, the digital platform 'NeuronUP' has been selected as the digital environment for cognitive stimulation and executive function training (see Fig. 2). NeuronUP is a cognitive rehabilitation and stimulation platform hosted on a cloud system, allowing access from any device via a web login [50]. In a systematic review by Guerrero & García [28], NeuronUP was analyzed and compared with other neuropsychological rehabilitation platforms. The authors found that NeuronUP had strengths in adaptability, flexibility, evidence of effectiveness, data management, and treatment, receiving a rating of 7.14 out of 10.

Launched in 2012, NeuronUP features a vast repository of digital activities designed to customize sessions according to the needs of each participant. These activities are contextualized to daily life situations related to functionality variables, ensuring ecological validity. They are based on strong theoretical frameworks and principles of neuropsychological habilitation and rehabilitation [50]. Many activities mimic paradigms similar to classic tasks (e.g., go/no go, Flanker, Stroop, among others). The platform consists of two sections:

(1) Activity Manager: It contains categorized material for conducting sessions.
(2) Results Manager: It stores the results of all activities performed by participants.

The activities adapt to the child's performance, increasing or decreasing in difficulty according to the child's needs. They are divided into three types: generators, games, and sheets.

- Generators are unlimited and ecological activities that prevent users from memorizing the exercise, encouraging them to focus on the process by creating endless versions of the same activity. These can be executed digitally or on paper and are highly customizable to facilitate the introduction of meaningful stimuli.
- Games are activities organized into different levels of difficulty, allowing users to advance or regress automatically based on their successes or errors. They can also be customized to suit each user's needs.
- Sheets are activities organized by levels of difficulty. They can be worked with correction, meaning the platform corrects the user after each action, or in a free manner, where the platform does not provide feedback until the end of the exercise, not indicating whether the user is right or wrong during the activity.

The platform includes various intervention areas such as cognitive functions, activities of daily living, and social skills. For the purposes of the current research, activities were selected to train executive functions (working memory, planning, flexibility, inhibition) and attention.

**Fig. 3:**
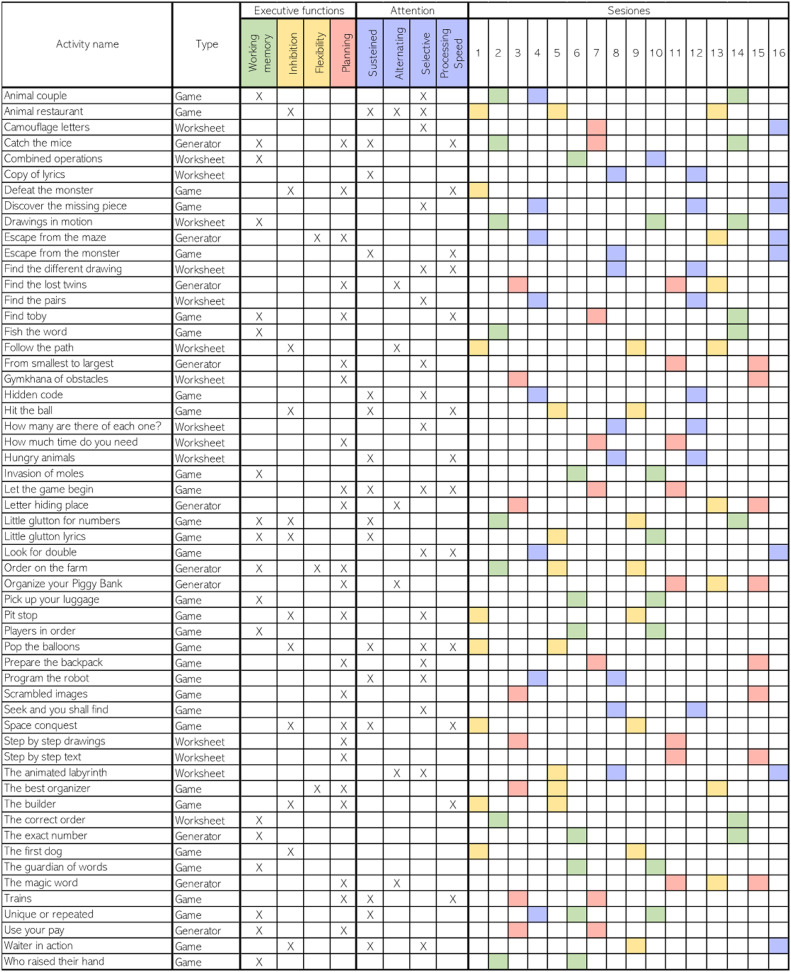
Selected activities and structure of digital sessions.

The researchers reviewed the available activities, and after a pilot exercise, 54 activities are selected and distributed across the 16 sessions. Each session consists of 8 activities, each programmed for 5 min, for a total of 40 min per session. Although each digital activity contributes to the training of various cognitive functions, activities with an emphasis on one cognitive process are selected for each session. Accordingly, the 16 sessions are distributed with the following emphasis: 4 sessions for working memory, 4 sessions for planning, 4 sessions for attention, and 4 sessions for inhibitory control and cognitive flexibility (the latter processes are integrated due to the lower number of available activities). Fig. 3 presents the selected digital activities from the NeuronUP platform, differentiating by type of activity (game, worksheet, and generator), the cognitive processes they train, and the session in which they are introduced.

• **Control group**

For the study, an active control group is selected. One benefit of an active control group is that it helps control for positive beliefs participants may have about the intervention's use, thereby reducing biases. It also enables blinding of participants who are not informed about which intervention is the study's target [51].

**Fig. 4:**
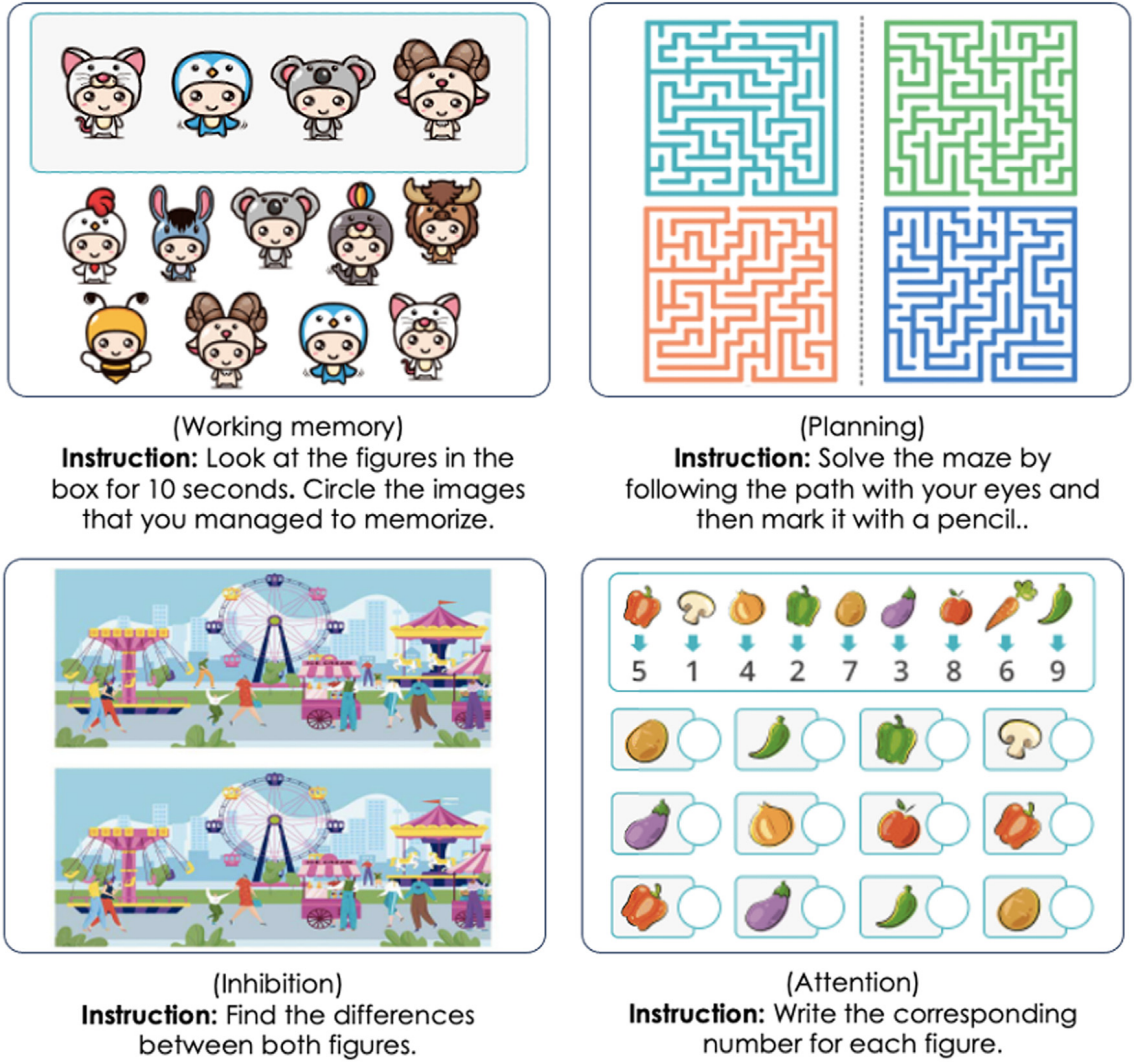
Example activities from eCognitiva.

***Source***: personal compilation based on www.ecognitiva.com.

The active control group will receive cognitive training based on paper-and-pencil exercises. This intervention has the same frequency and duration as the experimental group's intervention, with 2 sessions per week for 8 weeks. The intervention for the active control group will follow the same structure and objective as the experimental group's intervention. Therefore, the paper-and-pencil exercises selected for each session aims to train the same cognitive processes as the experimental group's sessions. Out of the 16 sessions, 4 emphasize working memory, 4 emphasize planning, 4 emphasize attention, and the remaining 4 emphasize flexibility and inhibition, as shown in Fig. 4.

For the paper-and-pencil sessions, freely available activities are selected. One of the sources is the cognitive stimulation worksheets and exercises from the eCognitiva.com website (See Fig. 4). This website offers workbooks focused on different cognitive domains such as attention, memory, language, and arithmetic (eCognitiva, n.d.). While the website provides materials for children [52] and adults with cognitive impairment [53], these materials can easily be adapted for other populations. Therefore, the researchers select them from the various available workbooks and distribute them across the different cognitive domains addressed in the study. Additionally, some paper-and-pencil activities designed by the researchers in previous studies [54] are adapted, as well as activities from other cognitive stimulation programs [55].

## Bias control

For bias control, we rely on the bias control tool for controlled trials from the Cochrane organization [56], which defines six domains of bias (see Table 2). We established actions to control the occurrence of the most common types of bias in non-clinical studies, such as selection bias, detection bias, performance bias, attrition bias, and reporting bias [20].

**Table 2:** Risk of Bias Control.

Domain	Mechanism to reduce bias risk
Selection bias	Subjects will be randomly assigned through an online randomization software, with this procedure being overseen by an external researcher.
Performance bias	The researchers responsible for the intervention will be blinded to the assignment and test results. Both the control and experimental groups will participate in an intervention; therefore, the participants will be blinded to the target intervention.
Detection bias	The researcher responsible for administering the pre-test and post-test measures will be blinded to the assignment.
Attrition bias	An intention-to-treat analysis approach will be used. Therefore, instead of excluding participants who did not complete the intervention, all participants will be included in the analysis. Synthetic data imputations will be conducted to project missing data, and subsequently, these results will be compared with the per-protocol analysis, which excludes missing data.
Reporting bias	The results will be published according to the objectives outlined in the protocol, regardless of whether the results confirm the proposed hypothesis or not.

## Statistical analysis plan

A hypothesis test will be conducted using Mixed-Model Analysis of Variance of Repeated Measures (RM-ANOVA), followed by Bonferroni corrections to examine differences between groups. In this analysis, pre-test and post-test evaluations will act as the within-subject factor (repeated measures), while the comparison between the experimental and control groups will serve as the between-subject factor. Partial eta squared will be used to quantify effect size, assuming variables meet the assumptions of homoscedasticity and normality for parametric tests; otherwise, non-parametric tests will be employed.

To analyze the relationship between participants' performance and various sociodemographic variables acting as potential confounders, multivariate linear regression analysis will be conducted for numerical variables, and logistic regression analysis will be applied for categorical variables.

## Ethical considerations

The study is registered with the Vice-Rectorate of Research-Creation, Innovation, Extension, and Social Projection of University of Tolima under code 50,119. The randomized controlled trial complies with the ethical parameters defined by the Helsinki Declaration [1], as well as the norms for health research established by the Colombian Ministry of Health [2] in Resolution 8430. Parents and guardians of the participating children will provide informed consent. This randomized controlled trial has been approved by the bioethics committee of University of Tolima, documented in act 10 of December 13th, 2022.

## Protocol validation

This article presents the protocol of a randomized controlled trial aimed at evaluating the effects of computerized cognitive training on executive functions and attention in school-aged children, compared to traditional pencil-and-paper cognitive training. The study is relevant and pertinent to the academic community for several reasons: 1) It contributes to the discussion on integrating cognitive training into school activities to support children's cognitive development. 2) It will help consolidate evidence on the impact of computerized cognitive training and pencil-and-paper training in typically developing school-aged children. 3) It explores whether the use of technological interventions in cognitive training yields different impacts compared to non-digital interventions. 4) It assesses the feasibility of integrating cognitive training programs into primary school curricula. 5) The designed protocol aims to ensure the quality of the collected evidence through thorough bias control. Among the measures to be implemented for bias control are blinding of evaluators to assignment, blinding of researchers responsible for the intervention to subject evaluation, blinding of participants to the target intervention, and randomization of subjects to both study arms. Synthetic data imputations will be employed to handle missing data in the study, opting for intention-to-treat analysis.

## Limitations

One of the limitations of the study is its reliance on school settings, which may be subject to changes in the institutional school calendar and technological constraints such as availability of electronic devices and access to high quality internet. Additionally, the use of a commercial platform poses another limitation, as the accessibility of such technological resources for cognitive training in educational institutions may be limited. However, the study aims to provide evidence supporting the development of new digital learning resources that could potentially be made openly available to teachers and education professionals.

## CRediT author statement

**Carolina Robledo-Castro:** conceptualization, methodological design, intervention design, selection of measurement instruments, analysis plan, protocol writing. **Gimena Rocio Ramírez Suárez:** conceptualization, intervention design, protocol writing. **Luz Helena Rodriguez-Rodriguez:** conceptualization, background review, methodological design, protocol writing, review, edition.

## Declaration of competing interests

The authors declare that they have no known competing financial interests or personal relationships that could have appeared to influence the work reported in this paper.

## Data Availability

Data will be made available on request.

## Appendix Supplementary materials

Image, application 1
